# Hybrid TRUS/CT with optical tracking for target delineation in image-guided adaptive brachytherapy for cervical cancer

**DOI:** 10.1007/s00066-020-01656-2

**Published:** 2020-07-03

**Authors:** Stéphanie Smet, Nicole Nesvacil, Johannes Knoth, Alina Sturdza, Dina Najjari-Jamal, Filip Jelinek, Gernot Kronreif, Richard Pötter, Joachim Widder, Christian Kirisits, Maximilian P. Schmid

**Affiliations:** 1 Department of Radiation Oncology, Comprehensive Cancer Center, Medical University of Vienna, Vienna, Austria; 2 Department of Radiation Oncology, General Hospital Turnhout, Turnhout, Belgium; 3 Christian Doppler Laboratory for Medical Radiation Research for Radiation Oncology, Medical University of Vienna, Vienna, Austria; 4 Department of Radiation Oncology, Institut Català d’Oncologia, Barcelona, Spain; 5 Austrian Center for Medical Innovation and Technology, Wr. Neustadt, Austria; 6 Department of Radiation Oncology, Comprehensive Cancer Center, General Hospital of Vienna, Medical University of Vienna, Währinger Gürtel 18–20, 1090 Vienna, Austria

**Keywords:** Locally advanced cervical cancer, Image-guided adaptive brachytherapy, Transrectal ultrasound, Contouring, MRI

## Abstract

**Objective:**

To prospectively compare the interobserver variability of combined transrectal ultrasound (TRUS)/computed tomography (CT)- vs. CT only- vs. magnetic resonance imaging (MRI) only-based contouring of the high-risk clinical target volume (CTV_HR_) in image-guided adaptive brachytherapy (IGABT) for locally advanced cervical cancer (LACC).

**Methods:**

Five patients with LACC (FIGO stages IIb–IVa) treated with radiochemotherapy and IGABT were included. CT, TRUS, and T2-weighted MRI images were performed after brachytherapy applicator insertion. 3D-TRUS image acquisition was performed with a customized ultrasound stepper device and software. Automatic applicator reconstruction using optical tracking was performed in the TRUS dataset and TRUS and CT images were fused with rigid image registration with the applicator as reference structure. The CTV_HR_ (based on the GEC-ESTRO recommendations) was contoured by five investigators on the three modalities (CTV_HR__CT, CTV_HR__TRUS-CT, and CTV_HR__MRI). A consensus reference CTV_HR__MRI (MRIref) was defined for each patient. Descriptive statistics and overlap measures were calculated using RTslicer (SlicerRT Community and Percutaneous Surgery Laboratory, Queen’s University, Canada), comparing contours of every observer with one another and with the MRIref.

**Results:**

The interobserver coefficient of variation was 0.18 ± 0.05 for CT, 0.10 ± 0.04 for TRUS-CT, and 0.07 ± 0.03 for MRI. Interobserver concordance in relation to the MRIref expressed by the generalized conformity index was 0.75 ± 0.04 for MRI, 0.51 ± 0.10 for TRUS-CT, and 0.48 ± 0.06 for CT. The mean CTV_HR__CT volume of all observers was 71% larger than the MRIref volume, whereas the mean CTV_HR__TRUS-CT volume was 15% larger.

**Conclusion:**

Hybrid TRUS-CT as an imaging modality for contouring the CTV_HR_ in IGABT for LACC is feasible and reproducible among multiple observers. TRUS-CT substantially reduces overestimation of the CTV_HR_ volume of CT alone while maintaining similar interobserver variability.

## Introduction

In image-guided adaptive brachytherapy (IGABT) of locally advanced cervical cancer (LACC), magnetic resonance imaging (MRI) with applicator in place is currently considered as the gold standard for tumor visualization and dose optimization [1–9]. MRI is, however, not always available in centers with fewer resources, which is why computed tomography (CT) has been evaluated as alternative, as most radiotherapy departments are equipped with a CT scanner [10]. Contouring on CT enables excellent visualization of the applicator and organs at risk (OAR), but also generally leads to an overestimation of the target volume compared to MRI [10].

Increasing evidence shows that transrectal ultrasound (TRUS) might be a more promising and inexpensive alternative. TRUS is extensively used in prostate brachytherapy, but apart from tumor assessment and confirming tandem placement, it has not been generally adopted in LACC brachytherapy to date [11]. First studies investigating TRUS for target definition in LACC brachytherapy indicated that TRUS appears non-inferior to MRI for assessing the high-risk clinical target volume (CTV_HR_) dimensions [12, 13]. As TRUS and CT are widely available in radiotherapy departments, combining their assets could possibly provide an equivalent for MRI in contouring target volumes in IGABT for LACC. A clinical workflow for combined TRUS/CT treatment planning in LACC brachytherapy has already been successfully simulated in a patient [14].

If TRUS is further developed as a treatment planning modality, a TRUS/CT-based target delineation protocol should be developed to allow for volumetric TRUS-based contouring in routine clinical practice. As ultrasound is known for its observer dependence and previous studies on TRUS for LACC brachytherapy were performed only with a single observer, further research with multiple observers is mandatory as a key step towards development of TRUS-based brachytherapy. The objective of this analysis was to investigate the feasibility of combined TRUS/CT-based contouring using a dedicated prototype system for LACC brachytherapy and to analyze interobserver variability for CTV_HR_ within a prospective pilot study with multiple observers.

## Methods

Five patients with squamous cell carcinoma of the uterine cervix treated with curative intent were included in this prospective pilot study (EK no. 1998/2014). Two patients had FIGO stage IIB disease, three had stage IIIC2 (local: IIB, IIIB, IVA, respectively; FIGO version 2018 [15]). All patients underwent a staging MRI at diagnosis. Treatment consisted of external beam radiation therapy (EBRT) with concomitant cisplatin and IGABT. During EBRT, the elective clinical target volume (CTV) received 45 Gy in 1.8-Gy fractions, with a simultaneously integrated boost to pathologic lymph nodes to 55–57.5 Gy in 2.2–2.3-Gy fractions. MRI-based IGABT was performed at the end or after EBRT in two applications of two fractions each, using high-dose-rate brachytherapy with a planning aim of delivering a D90 >85 Gy EQD2_10_ to the CTV_HR_ (EBRT + IGABT). A tandem-ring applicator (“Vienna-type” applicator, Elekta, Sweden) without or with (straight, oblique, or freehand) interstitial titanium needles was used.

### Imaging modalities for IGABT

TRUS, CT, and MRI were performed after applicator placement. A Somatom Plus S scanner was used for CT (Siemens, Erlangen, Germany), in 2‑mm slice intervals without contrast medium. T2-weighted MRI was performed with a 0.35 T system (Magnetom C, Siemens) with 5‑mm slice thickness, acquiring axial, para-axial, sagittal, and coronal images.

### TRUS system

TRUS was performed with a dedicated prototype system developed for LACC brachytherapy allowing for 3D-TRUS imaging with automatic applicator reconstruction by optical tracking of the applicator (MedCom, Germany; Elekta; ACMIT, Austria). The main principle of the system is that the TRUS probe and the applicator are fixed to each other by a dedicated positioning arm and that tracking tools are positioned at specific reference geometries, which can be identified by the optical tracking system. Overall, the prototype consists of (1) a mobile cart with a PC equipped with (2) an imaging software (GynUS v2.0, MedCom); (3) a 5–10-MHz integrated TRUS transducer (BiopSee, MedCom) with a biplanar probe (BIPC6.5/10/128, BIPL7.5/70/128), mounted to (4) a stepper unit (ECRM, Elekta) with affixed (5) tracking tools (ACMIT) containing factory NDI trackers (single-faced rigid bodies) tracked by (6) a ceiling-mounted Polaris Spectra system (NDI, Canada); and (7) multi-DOF passive positioning arms connecting the applicator to the stepper unit (Baitella, Switzerland), and connecting the complete imaging unit to the operating table’s side rail (MFA, iSYS Medizintechnik, Austria). Further technical details have been published elsewhere [16, 17].

### TRUS imaging and tracking procedure

**Fig. 1:**
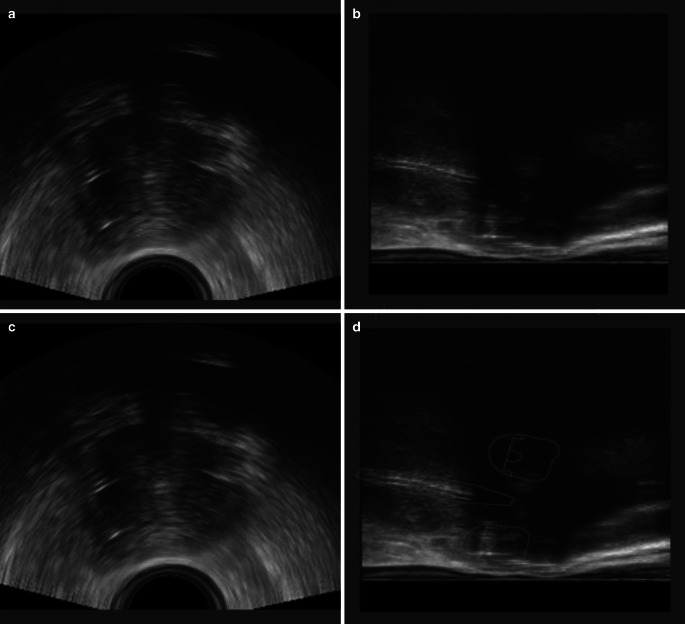
Example transrectal ultrasound (TRUS) images of a patient with FIGO IIB cervical carcinoma at time of brachytherapy with the applicator in place: **a** axial view; **b** sagittal view; **c** axial view with reconstructed applicator; **d** sagittal view with reconstructed applicator

After applicator insertion, the TRUS probe with the stepper unit and loose MFA arms was placed manually in the rectum at the optimal depth and angle according to the position and flexure of the uterus/applicator. The MFA arm was locked and the stepper unit subsequently connected to the applicator with the second positioning arm. Image acquisition was performed during automated rotation of the TRUS probe, generating a 3D volume using the longitudinal array of the probe from the level of the ring applicator to the fundus of uterus, if reachable. Optical tracking of the applicator was based on a set of retroreflective marker spheres mounted on both the TRUS probe and the applicator. The relative position of the two components was tracked with a stereo camera (Polaris, NDI, Canada). The system has already been described by Jelinek et al. [17]. Using spatial information from the tool tracking system, automatic applicator reconstruction using applicator library models was performed ([14]; Fig. 1). The reconstruction process was verified within a plausibility check based on several reference structures (tandem, posterior curvature of the ring, rotation of the ring as indicated by the holes, interstitial needles) and fine-tuned, if necessary. The positioning arms were then unlocked and the imaging unit was removed. The resulting TRUS image volumes were rigidly registered to the post-implant CT using the applicator as a reference coordinate system as previously described [14].

All further image registration and contouring operations were performed in the Oncentra brachytherapy treatment planning system (TPS; Elekta).

### Contouring protocol and image analysis

Five investigators were available for analysis. The investigators were all radiation oncologists with experience in contouring brachytherapy target volumes on CT and MRI in LACC. For TRUS-based contouring, only one observer was experienced.

All investigators were given an introductory course on TRUS contouring by the experienced investigator and two test cases were contoured by all investigators. After approval of these cases, the study cases could be contoured. Investigators were provided with the following material: clinical information of each patient, a pelvic MRI at diagnosis, and a 3D clinical drawing of the gynecological examination at diagnosis and at the time of brachytherapy [18].

**Fig. 2:**
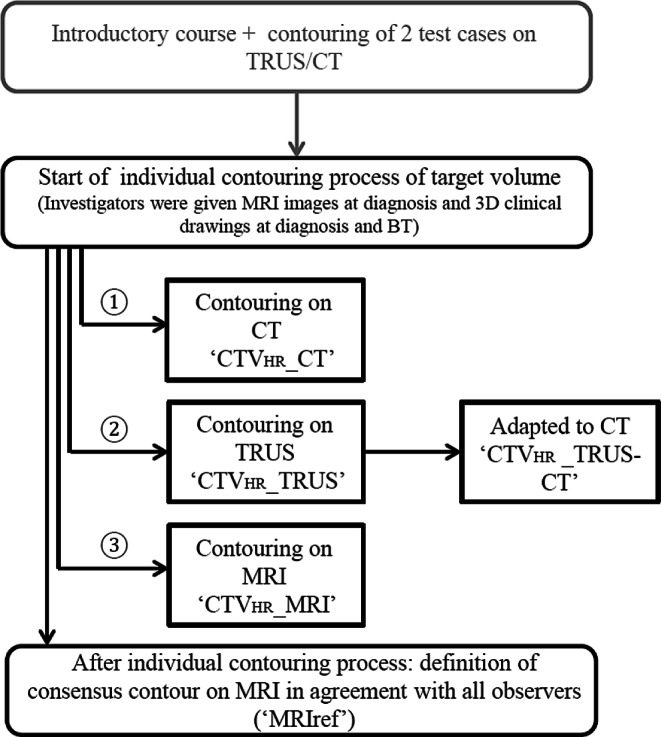
Flowchart of contouring protocol. *TRUS* transrectal ultrasound, *CT* computed tomography, *BT* brachytherapy, *MRI* magnetic resonance imaging, *MRIref* reference high-risk clinical target volume (*CTV*_*HR*_) on MRI

All investigators contoured each case independently following the workflow defined in Fig. 2. First, the CTV_HR_ was contoured on CT (CTV_HR_ _CT) according to the consensus guidelines for CT-based brachytherapy, defined by the complete mass (cervical + parametrial) at intermediate density (grey/white). The CTV_HR_ height on CT was determined by the GTV height on the MRI at diagnosis [10].

Secondly, the CTV_HR_ was contoured on TRUS (CTV_HR__TRUS), defined by a hypoechogenic mass (cervical + parametrial) on grey-scale imaging [13]. After contouring this preliminary target volume on TRUS, the structures were resampled to CT and adapted according to the OAR depicted on CT (CTV_HR__TRUS-CT). The CTV_HR_ height on TRUS-CT was determined by the GTV height on the MRI at diagnosis.

Next, the CTV_HR_ was contoured on MRI (CTV_HR__MRI) according to the recommendations for MR-based brachytherapy as the whole cervix and any residual disease at time of brachytherapy including “grey zones” [19, 20].

Finally, a reference contour on MRI (MRIref) was generated for each patient, attained by reaching consensus amongst all observers.

### Analysis of contouring deviations

For each imaging modality, the maximum width (maximum latero-lateral diameter found in all para-axial slices), the maximum thickness (maximum antero-posterior diameter found in all para-axial slices), the maximum height (maximum cranio-caudal diameter found in all sagittal slices), and the total volume of each CTV_HR_ from every observer was measured for each patient.

**Fig. 3:**
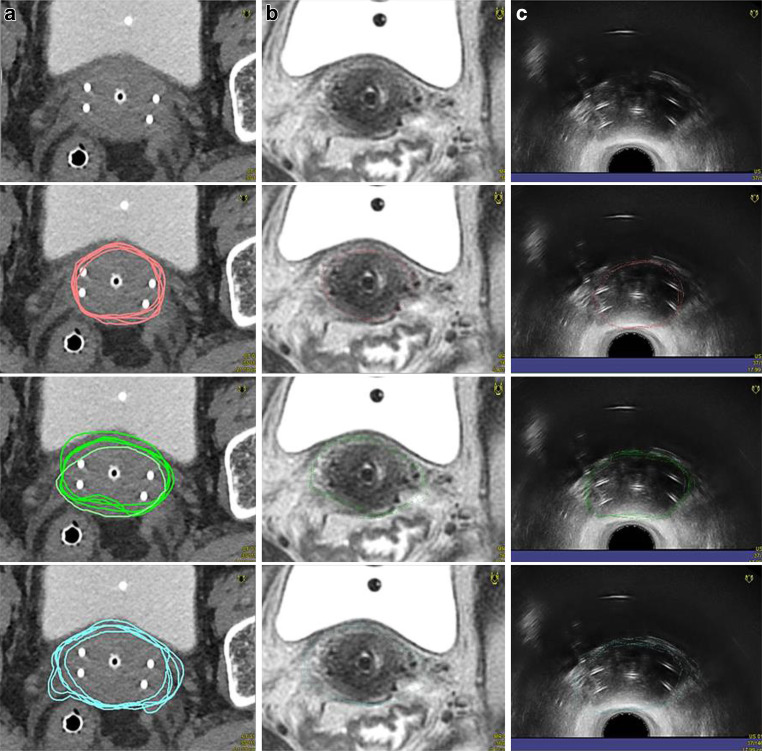
Example contours of a patient with FIGO IIB cervical carcinoma at time of brachytherapy with the applicator in place on computed tomography (CT) (**a**), magnetic resonance imaging (MRI) (**b**), and transrectal ultrasound (TRUS) (**c**). Contours from all observers are projected on the CT dataset: MRI contours in *pink*, TRUS contours in *green*, CT contours in *blue*

CT and MRI images were automatically registered with TRUS-CT images based on the applicator position, serving as a common reference coordinate system defined on the TPS [21]. Finally, all contours were superimposed on a single CT image set per patient, including all contours from all imaging modalities from all observers, allowing for direct comparison to each other (Fig. 3). To assess interobserver variations, all images and contours were exported to the SlicerRT software system, version 4.5.0‑1 (SlicerRT Community and Percutaneous Surgery Laboratory, Queen’s University, Canada) [22]. Contours of every observer were compared with one another and with the MRIref.

The coefficient of variation (COV) was defined as the standard deviation divided by the mean value and is larger with increasing variability (more dispersion in the data). A generalized conformity index (CIgen) was defined as the ratio of the sum of all overlapping volumes between pairs of observers and the sum of all overlapping and all additional volumes between the same pairs [23]. A CIgen of 1 indicates a total overlap, while 0 means there is no concordance. Descriptive statistics, mean differences between the groups, and a paired t‑test were calculated. Statistics were performed with Excel 2010 software (Microsoft, Washington). A *p*-value <0.05 was considered as statistically significant.

## Results

The proposed workflow of hybrid TRUS-CT with optical tracking of the applicator was successfully applied. 3D-TRUS imaging, automatic applicator reconstruction, volumetric contouring on TRUS, and image fusion with CT were feasible in all patients. All observers were able to contour the CTV_HR_ on TRUS-CT for all patients, with an acceptable agreement between the observers (CIgen: 0.66 ± 0.08; COV: 0.10 ± 0.04).

The mean maximum width of the CTV_HR_ was 55 mm ± 8 mm, 52 mm ± 6 mm, and 64 mm ± 6 mm, and the mean volume was 46 cm^3^ ± 9 cm^3^, 43 cm^3^ ± 10 cm^3^, and 67 cm^3^ ± 9 cm^3^ on TRUS-CT, MRI, and CT, respectively. The mean CIgen was 0.66 ± 0.08, 0.75 ± 0.04, and 0.66 ± 0.06, and the mean COV was 0.10 ± 0.04, 0.07 ± 0.03, and 0.18 ± 0.05 for TRUS-CT, MRI, and CT, respectively (Table 1).

**Table 1:** Mean maximum width (mm), maximum thickness (mm), maximum height (mm), and volume (cm^3^) with respective ranges (min–max); generalized conformity index; and coefficient of variation from all observers for contours on CT, TRUS-CT, and MRI

		Patient 1	Patient 2	Patient 3	Patient 4	Patient 5	Mean (SD)
*CT*	Width	75 (64–82)	58 (56–61)	62 (54–66)	63 (56–70)	62 (55–72)	–
	Height	58 (53–63)	52 (46–57)	52 (41–58)	64 (54–72)	68 (62–76)	–
	Thickness	40 (38–42)	44 (39–49)	43 (41–45)	34 (25–39)	34 (30–37)	–
	Volume	82 (49–102)	59 (47–64)	68 (46–79)	56 (36–63)	71 (40–85)	67 (9)
	CIgen	0.60	0.73	0.71	0.65	0.59	0.66 (0.06)
	COV	0.23	0.10	0.17	0.18	0.24	0.18 (0.05)
*TRUS-CT*	Width	69 (62–71)	51 (43–57)	57 (54–58)	49 (45–51)	47 (45–50)	–
	Height	55 (38–62)	46 (45–50)	47 (41–54)	59 (51–64)	65 (54–73)	–
	Thickness	34 (32–39)	35 (30–39)	37 (33–42)	31 (25–36)	29 (24–32)	–
	Volume	60 (44–71)	43 (40–43)	51 (45–60)	35 (29–39)	40 (30–48)	46 (9)
	CIgen	0.60	0.77	0.71	0.65	0.59	0.66 (0.08)
	COV	0.14	0.03	0.10	0.11	0.13	0.10 (0.04)
*MRI*	Width	64 (60–73)	47 (43–50)	48 (46–50)	53 (49–60)	48 (45–49)	–
	Height	53 (48–56)	54 (51–61)	44 (41–46)	49 (45–52)	44 (41–46)	–
	Thickness	42 (41–44)	34 (32–36)	37 (36–38)	21 (17–23)	37 (36–38)	–
	Volume	59 (53–65)	40 (36–47)	43 (41–45)	30 (26–34)	43 (40–45)	43 (10)
	CIgen	0.74	0.72	0.81	0.73	0.78	0.75 (0.04)
	COV	0.08	0.10	0.03	0.09	0.05	0.07 (0.03)

*CT* computed tomography, *TRUS-CT* transrectal ultrasound combined with computed tomography, *MRI* magnetic resonance imaging, *CIgen* generalized conformity index, *COV* coefficient of variation, *SD* standard deviation

No significant difference was observed in the mean maximum width, height, thickness, or volume on TRUS-CT compared to MRI. The mean maximum width and volume were significantly larger on CT compared to MRI (Tables 3 and 4).

**Fig. 4:**
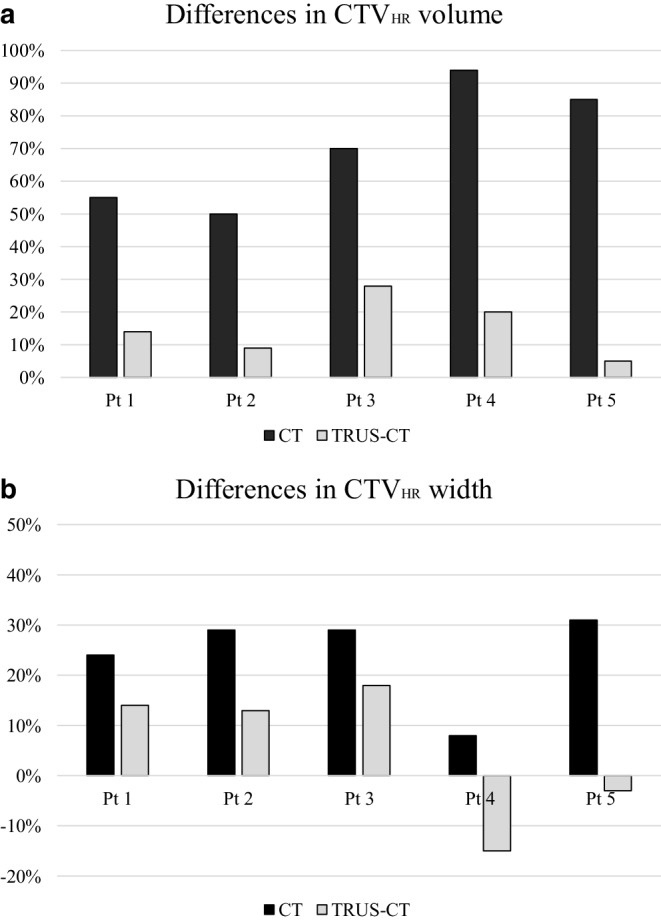
Relative over-/underestimation of the mean volume of the high-risk clinical target volume (*CTV*_*HR*_; **a**) and mean width of the CTV_HR_ (**b**) on CT (*black*) and on TRUS-CT (*grey*) compared to the reference CTV_HR_ on MRI for all patients. *Pt* patient, *CT* computed tomography, *TRUS-CT* transrectal ultrasound combined with computed tomography

The mean volume of the CTV_HR_ was 15% larger on TRUS-CT (14%, 9%, 28%, 20%, and 5% for patients 1–5, respectively) and 71% larger than on CT (55%, 50%, 70%, 94%, and 85% for patients 1–5, respectively) than on the MRIref volume (Fig. 4a). The mean maximum width of the CTV_HR_ was 5% larger on TRUS-CT (14%, 13%, 18% larger for patients 1, 2, and 3; 15% and 3% smaller for patients 4 and 5, respectively) and 24% larger on CT (24%, 29%, 29%, 8%, and 31% for patients 1–5, respectively) than on the MRIref contour (Fig. 4b). The CIgen was 0.51 ± 0.1, 0.75 ± 0.04, and 0.48 ± 0.06 for all TRUS-CT, MRI, and CT contours, respectively, in relation to the MRIref contour (Table 2).

**Table 2:** Mean generalized conformity index for CT, TRUS-CT, and MRI in relation to MRI reference volume

CIgen	Patient 1	Patient 2	Patient 3	Patient 4	Patient 5	Mean (SD)
CT–MRIref	0.49	0.54	0.53	0.48	0.39	0.48 (0.06)
TRUS–MRIref	0.51	0.57	0.63	0.49	0.38	0.51 (0.10)
MRI–MRIref	0.73	0.70	0.80	0.75	0.78	0.75 (0.04)

*CT* computed tomography, *TRUS-CT* transrectal ultrasound combined with computed tomography, *MRI* magnetic resonance imaging, *CIgen* generalized conformity index, *SD* standard deviation, *MRIref* reference high-risk clinical target volume (CTV_HR_) on MRI

The differences in mean maximum width, height, and volume were significantly smaller for TRUS-CT compared to MRIref than for CT compared to MRIref (Tables 3 and 4).

**Table 3:** Differences (*p-*values) in mean maximum width, thickness, height, and volume on TRUS-CT vs. MRI and CT vs. MRI

	Width	Thickness	Height	Volume
TRUS-CT vs. MRI	0.32	0.78	0.31	0.21
CT vs. MRI	<0.01	<0.01	<0.01	<0.01

*TRUS-CT* transrectal ultrasound combined with computed tomography, *CT* computed tomography, *MRI* magnetic resonance imaging

**Table 4:** Differences (*p*-values) in mean maximum width, thickness, height, and volume between TRUS-CT compared to MRIref and CT compared to MRIref

	Width	Thickness	Height	Volume
TRUS-CT vs CT	0.01	0.16	<0.01	<0.01

*TRUS-CT* transrectal ultrasound combined with computed tomography, *CT* computed tomography, *MRI* magnetic resonance imaging, *MRIref* reference high-risk clinical target volume (CTV_HR_) volume on MRI

## Discussion

Contouring uncertainties due to poor target volume visualization have a substantial impact on tumor coverage and dose to OAR. MRI is currently considered the gold standard for target volume contouring in IGABT in LACC because of the high tissue contrast in pelvic anatomy [10, 20], but is not always accessible due to its cost. Therefore, CT could serve as a plausible alternative, but literature on the comparison of CT- and MRI-based contouring showed a substantial overestimation of CTV_HR_ volume on CT [18, 24, 25]. Contouring of the extent of parametrial invasion remains challenging due to lacking soft tissue contrast on CT, specifically in large tumors with extensive invasion [24]. Recently, TRUS has been considered as an imaging modality for IGABT, since it provides excellent soft tissue contrast and has already proven its advantages in prostate cancer brachytherapy [26]. Schmid et al. suggested implementing TRUS in the pre- and intraoperative setting to assess the CTV_HR_ before applicator insertion, for preplanning purposes, and for TRUS-guided tandem and needle insertion [12]. A comparison of the target volume dimensions between TRUS and MRI showed no statistically significant difference for the CTV_HR_ width, indicating the high potential of TRUS for IGABT in LACC [13]. To integrate TRUS into IGABT and to outweigh subsequent limitations, a workflow for combined TRUS-CT was established by Nesvacil et al. [14], showing that TRUS-CT-based volumetric contouring and treatment planning is feasible and may be clinically comparable to the MRI-based approach.

In this report, volumetric contouring for LACC on 3D-TRUS with multiple observers was investigated for the first time. The previously described workflow for TRUS-CT treatment planning proved feasible within this prospective pilot study and all observers managed to follow a contouring protocol for combined TRUS-CT-based CTV_HR_ contouring. A dedicated prototype comprising standard equipment from different fields including a standard TRUS probe, fixation devices, and an optical tracking system were safely applied. Particularly the TRUS probe positioning and imaging process using a table-mounted flexible stepper unit in combination with fixation devices was a substantial improvement in comparison to the system used in our previous studies. 3D-TRUS imaging with the applicator in place could therefore be applied for all patients in this report. It appears that with such a system, the previously reported substantial proportion (≈20%) of patients not suitable for TRUS imaging with applicator could be significantly reduced. Since the location of the applicator, particularly the ring-component, can’t be defined on TRUS with reasonable accuracy for subsequent treatment planning, optical tracking of the applicator was implemented in the system to allow for automatic applicator reconstruction in the TRUS dataset. This is a crucial step within the proposed workflow to enable further image fusion with CT and treatment planning. Extensive phantom and clinical tests were performed in advance to learn and improve the tracking procedure; however, optical tracking is sensitive to various interferences and shows limitations in daily clinical practice for LACC brachytherapy in its current form. Nevertheless, automatic applicator reconstruction using applicator library models was successfully performed, with some minor manual adjustments, in all patients in this study. Further optimization and investigation of alternative tracking modalities, such as electromagnetic or mechanical tracking, are necessary.

In accordance with our previous study, the mean maximum width of the CTV_HR_ on TRUS-CT was smaller than on CT alone and comparable to MRI, indicating the reproducibility of these previous findings with multiple observers. Furthermore, combining TRUS and CT reduced the mean CTV_HR_ volume by approximately one third, reduced the mean COV from 0.18 to 0.10, and slightly improved the CIgen from 0.48 to 0.51 compared with the MRIref volume. As expected, the variation for the MRI contours was smaller (mean COV = 0.1 and mean CIgen = 0.75 between all observers and with regard to the MRIref volume), whereas the mean CTV_HR_ volume was similar between TRUS-CT (46 cm^3^) and MRI (43 cm^3^). These results are in line with the literature on interobserver variations in target delineation of IGABT in LACC: Petric et al. reported a CIgen of 0.78 for MRI-based contouring based on 13 cases and two observers [27], Viswanthan et al. reported a CIgen of 0.48 for CT-based contouring based on 3 cases and 23 observers [24], and Pötter et al. reported a CIgen of 0.54 for CT-based contouring with support of a pre-brachytherapy MRI scan [25]. Performance of TRUS appears to have a similar impact on interobserver variation as a pre-brachytherapy MRI scan, but with the advantage of having the applicator already in place as a reference structure. In addition, Mahantshetty et al. recently investigated the use of TRUS assistance for CT-based target contouring by using measurements of reference points in relation to the cervical canal instead of full volumetric image fusion, and did not observe major deviations compared to MRI-based contouring [28].

The limitations of TRUS are the limited field-of-view and applicator-induced artefacts, implying that parts of the target volume are hardly or not visible on TRUS images alone (Fig. 1). Especially the limited depth of insertion of the transrectal probe in the presence of the applicator hampers depiction of the most cranial parts of the uterus, as was the case for the presented cases. To reach a consensus amongst observers, the initial GTV height as seen on the diagnostic MRI was adopted as the CTV_HR_ height on CT or TRUS-CT. Also, the anterior border of the CTV_HR_ adjacent to the bladder was more difficult to assess, due to the distance from the probe and acoustic shadowing by the applicator, resulting in a wider range in the maximum thickness on TRUS-CT compared to MRI. The presence of the TRUS probe can also cause compression of the uterus, leading to a reduced thickness of the CTV_HR_ as shown in our previous study [13]. In our present study, however, no significant difference between TRUS-CT and MRI was observed. The posterior and lateral parts of the cervix were well defined on TRUS, resulting in a consistently comparable range in the maximum width on TRUS-CT and MRI. In two patients, the maximum width on TRUS-CT was smaller compared to the MRIref contour (15% and 3% in patients 4 and 5, respectively), which should be interpreted with caution as the absolute differences are minor (8 mm ± 2 mm for patient 4 and 1 mm ± 2 mm for patient 5), but needs to be taken seriously, as this could result in a potential topographical miss by TRUS-CT. A random geometric uncertainty of up to 2 mm between contours from different modalities was noticed during conformity analysis. This may be reducible by further technical refinement of the applicator-based TRUS/CT registration methods. The dosimetric impact of the interobserver variations will be presented separately. In the present study, low-field MRI was used as planning modality. Although high-field MRI (1.5–3 T) MRI is more widely available, both are considered suitable for IGABT [20], and a previous report comparing target volume dimensions between TRUS and 1.5 T MRI showed similar results [13].

Another limitation of TRUS could be the operator dependence. Four out of five observers had very limited to no experience with TRUS-based contouring. Therefore, an introductory course into TRUS-based contouring was given and two training cases were contoured. Despite minimal training, excellent results with an acceptable interobserver variation could be achieved. Further improvement with increasing experience can be expected. Of note, while image interpretation and contouring were performed by multiple observers, TRUS image acquisition was performed by only one observer. The operator dependence of TRUS image acquisition is another possible source of variation, which was not addressed in the current study and has to be investigated separately. A similar learning curve as described for TRUS-based prostate brachytherapy can be assumed for TRUS in LACC brachytherapy [29, 30].

The present study covers interobserver contouring using TRUS-CT as one step further in the development of TRUS as a possible treatment modality in IGABT. However, other key questions remain to be answered, in particular regarding optimization of the imaging and tracking procedure, the performance of TRUS by multiple observers, and the actual treatment of patients by any form of TRUS-based brachytherapy within a clinical study.

## Conclusion

TRUS-CT-based contouring in IGABT for LACC with automatic applicator reconstruction by optical tracking is feasible after a minimal amount of training, is consistent amongst multiple observers, and leads to reduced volumes compared to CT alone. This prospective interobserver analysis could be the next step in the shift from TRUS being just an aid for tumor assessment and tandem placement verification during the procedure, to an accessible alternative to MRI for CTV_HR_ contouring.
